# Current hypertension epidemiology and contemporary approaches using the “Real-World Evidence Cycle” framework

**DOI:** 10.1038/s41440-025-02532-1

**Published:** 2026-01-09

**Authors:** Michihiro Satoh, Shingo Nakayama, Hiroki Nobayashi, Yutaro Iwabe, Seiya Izumi, Takahisa Murakami, Takuo Hirose, Hirohito Metoki

**Affiliations:** 1 Division of Public Health, Hygiene and Epidemiology, Faculty of Medicine, Tohoku Medical and Pharmaceutical University, Sendai, Japan; 2 Department of Preventive Medicine and Epidemiology, Tohoku Medical Megabank Organization, Tohoku University, Sendai, Japan; 3 Department of Pharmacy, Tohoku Medical and Pharmaceutical University Hospital, Sendai, Japan; 4 Department of Pathology and Laboratory Medicine, Cedars-Sinai Medical Center, Los Angeles, CA USA; 5 Division of Nephrology and Hypertension, Department of Internal Medicine, The Jikei University School of Medicine, Tokyo, Japan; 6 Center for Clinical Research Promotion and Development, Tohoku Medical and Pharmaceutical University Hospital, Sendai, Japan; 7 Department of Obstetrics and Gynecology, Tohoku University Graduate School of Medicine, Sendai, Japan; 8 Division of Aging and Geriatric Dentistry, Department of Rehabilitation Dentistry, Tohoku University Graduate School of Dentistry, Sendai, Japan; 9 Division of Nephrology and Hypertension, Faculty of Medicine, Tohoku Medical and Pharmaceutical University, Sendai, Japan; 10 Division of Integrative Renal Replacement Therapy, Faculty of Medicine, Tohoku Medical and Pharmaceutical University, Sendai, Japan; 11 Tohoku Institute for the Management of Blood Pressure, Sendai, Japan

**Keywords:** Hypertension, Epidemiology, Digital hypertension, Real-world data, Implemental hypertension

## Abstract

Hypertension is a major contributor to the global disease burden, affecting more than one billion individuals worldwide. Despite decades of recognition of its adverse health effects, hypertension control rates remain suboptimal. Epidemiology provides essential knowledge for understanding disease distribution and identifying risk factors at the individual, social, and environmental levels. Recent evidence emphasizes both traditional lifestyle determinants, including excess sodium intake, low potassium intake, obesity, physical inactivity, smoking, and alcohol consumption, and emerging contributors, such as gut microbiota dysregulation and infectious diseases. Among those, the urinary sodium-to-potassium ratio has gained attention as an important factor associated with hypertension risk. Additionally, social determinants of health, including socioeconomic disparities, neighborhood deprivation, and structural racism, exacerbate the risk of hypertension and impede its effective control. Environmental factors such as air pollution, extreme temperatures, and occupational stress further contribute to the complexity of hypertension epidemiology. Regarding contemporary epidemiological methodology, our proposed concepts of the “Bench and Real-World Cycle” and “Real-World Evidence Cycle” highlight the necessity of continuously integrating real-world evidence into practice. In addition to classical cohort studies, real-world data derived from electronic health records including health checkups and insurance claims data are indispensable tools for addressing previous research limitations. This multifaceted perspective will accelerate evidence-based epidemiological approaches for preventing and treating hypertension.

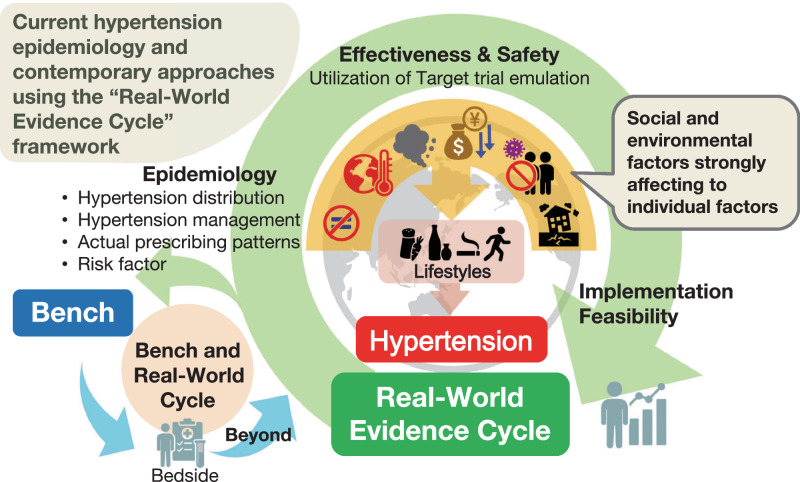

## Introduction

Hypertension is a major contributor to the global disease burden [1]. The number of patients with hypertension continues to increase because of an aging population, particularly in developed countries. Although the adverse effects of hypertension on the global burden of disease have been recognized for decades [2], hypertension remains a crucial target for improving public health. Accordingly, multiple regional and international guidelines have been developed worldwide to address this challenge [3–8]. In these guidelines, epidemiology provides the fundamental knowledge needed to understand hypertension and describe its distribution. This mini-review introduces the latest epidemiological evidence and proposes new concepts for utilizing real-world data to develop contemporary epidemiological approaches for future countermeasures.

## Hypertension statistics and burden

Globally, hypertension affected ~648 million people aged 30–79 years in 1990 and increased to 1.28 billion by 2019, suggesting that approximately one-third of adults have hypertension [9]. If current trends continue, the global burden is projected to reach ~1.5 billion people by 2030 and 2.0 billion people by 2050. In Japan, the number of adults with hypertension is estimated to be 43 million [10, 11], implying that nearly half of Japanese adults have hypertension. These estimations were based on blood pressure (BP) ≥ 140/ ≥ 90 mmHg as the definition for both hypertension diagnosis and BP control targets [9–11]. Meanwhile, lower BP cutoff points are gaining attention, as illustrated in the United States, where 130/80 mmHg serves as the diagnostic threshold for hypertension [4].

Reducing the risk for cardiovascular disease at lower BP levels will be a crucial challenge. A significantly increased risk of cardiovascular disease has been observed even in individuals with elevated BP (systolic/diastolic BP of 130–139/80–89 mmHg) in Japan [12–14]. According to the Evidence for Cardiovascular Prevention from Observational Cohorts in Japan (EPOCH-JAPAN), the population attributable fraction (PAF) of BP ≥ 130/80 mmHg for total cardiovascular disease mortality in patients without antihypertensive treatment was 38.6% [13]. A similar PAF estimation based on cardiovascular disease incidence was observed in another study conducted in Japan [14]. The Global Cardiovascular Risk Consortium recently reported that elevated BP and smoking are the two most influential modifiable risk factors for global longevity [15]. At an index age of 50 years, a systolic BP ≥ 130 mmHg shortened life expectancy in women and men by 1.7 and 1.8 years, respectively [15]. Moreover, it shortened the cardiovascular disease-free life expectancy by 1.3 and 1.8 years in women and men, respectively. Although the effect size appears modest compared to that of smoking or diabetes [15], the extremely high prevalence of elevated BP yields a profound population-level impact. Another estimate revealed that high systolic BP contributed to 10.85 million deaths in 2021 with the global population-attributable fraction of 15.99% [16]. Hypertension contributes to increased healthcare costs globally [17].

The relative cardiovascular risk due to high BP is more pronounced in younger populations [12–14]. However, because relatively short-term cardiovascular risks, such as 5- or 10-year risks, are very low in younger populations, healthcare providers sometimes have difficulty encouraging young individuals to modify their behavior. In this regard, lifetime risk estimates can be helpful tools because they represent the absolute risk during the remainder of one’s life [18]. Using lifetime risk estimation, we can provide young populations with high actual absolute risk estimates that they may face over their extended lifetimes [19–21].

## Hypertension risk factors at the individual level

Traditional risk factors for hypertension include unhealthy alcohol consumption, smoking, physical inactivity, unhealthy dietary patterns, and obesity [3–7, 16]. Table 1 summarizes the lifestyle-related risk factors highlighted in recent hypertension guidelines [3–7]. As a representative unhealthy dietary pattern, high sodium and low potassium intake are key targets in promoting a population-based approach to combat high BP. In this context, urinary sodium-to-potassium ratio is now the focus because its measurement is more convenient than sodium or potassium measurements and is associated with BP [22–28]. Furthermore, the urinary sodium-to-potassium ratio is more strongly associated with home BP than with office BP [29] and is reportedly associated with cardiovascular diseases, including subclinical heart failure [22, 30–32]. This is supported by the results showing that salt substitutes with reduced sodium levels and increased potassium levels lowered BP and reduced the risk of cardiovascular disease and all-cause mortality compared to regular salt [33]. The influence of salt sensitivity should be considered when assessing the association between salt intake and hypertension. Asian population may exhibit greater salt sensitivity, potentially due to “relative aldosterone excess” reflected in the high aldosterone-to-renin ratio, often accompanied by a low-renin profile [34–40]. This may be supported by the fact that primary aldosteronism is a common form of secondary hypertension in Asia [41–43].

**Table 1:** . Modifiable lifestyle-related hypertension risk factors

Category	Specific risk factor
Body weight	– Overweight/Obesity
Dietary factors	– High sodium intake (sodium/potassium ratio) – Low potassium intake – Lower calcium/magnesium intake – Unhealthy dietary patterns – Low dietary fiber – Alcohol consumption – Caffeine consumption – Sweetened beverages
Physical activity	– Low physical activity (low aerobic/ resistance exercise) – Sedentary behavior
Tobacco use	– Smoking – Passive smoking – Water pipe smoking – e-cigarettes
Psychosocial factors	– Chronic stress – Severe mental illness
Sleep disorders	– Poor sleep habits – Untreated or inadequately treated obstructive sleep apnea
General lifestyle/ others	– Unhealthy lifestyle overall – Unfavorable occupational environment – Gut microbiota dysbiosis

These factors are most commonly mentioned in the hypertension management guidelines [3–8]

Accumulating evidence suggests that gut microbiota dysregulation may contribute to the pathogenesis of hypertension [44, 45]. A cross-sectional study of Hong Kong Chinese adults demonstrated that differences in gut microbiota composition and gut microbiota-associated short-chain fatty acid profiles were associated with hypertension in women but not in men [46]. This suggests that dysregulation of the gut microbiota contributes to the sex differences underlying the pathology of hypertension. Another study reported that resistant hypertension is characterized by decreased microbial diversity, elevated Actinobacteria levels, and distinct metabolomic profiles involving glycerophospholipid metabolism [47]. A Japanese study suggested that gut microbiome variability may influence the renin-angiotensin system through various pathways [48]. These findings suggest that the gut environment could be a candidate target for hypertension management. Bowel movements may be a confounding or mediating factor for these associations because a longitudinal study demonstrated that the absence of daily bowel movements was associated with elevated day-to-day BP variability [49].

Infectious diseases can lead to hypertension. A retrospective study reported that hospitalized Coronavirus Disease-2019 (COVID-19) patients had a 2.23 times and non-hospitalized COVID-19 patients had a 1.52 times higher risk for development of persistent hypertension than influenza counterparts [50]. A cross-sectional study of 19 survivors with severe COVID-19 observed significantly elevated muscle sympathetic nerve activity, arterial stiffness, and impaired endothelial function after discharge for COVID-19 [51].

Genetic factors are unmodifiable but crucial for predicting hypertension. Children can inherit a parental history of hypertension, which contributes to the development of high BP in adolescence [52]. The use of polygenic risk scores has been explored for prediction [53–56]. The Tohoku Medical Megabank Community-Based Cohort Study recently highlighted the predictive value of polygenic risk scores, which capture the cumulative burden of hypertension-associated genetic variants, even in adults without a family history of hypertension [57, 58].

## Hypertension risk factors in the social and surrounding environment

**Fig. 1:**
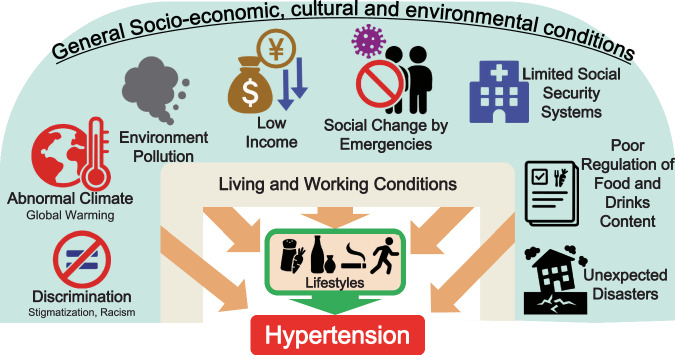
Possible social determinants influencing hypertension: a multilevel perspective. This framework was adapted from the Dahlgren-Whitehead rainbow model of the social determinants of health [59]

The “Social Determinants of Health (Rainbow Model)” by Dahlgren & Whitehead [59], along with the “Ecological Model” by McLeroy et al. [60], highlight that upstream factors such as socioeconomic conditions, social environment, and environment play a crucial role in the prevention and control of hypertension beyond individual lifestyle factors (Fig. 1).

The most concerning increases in hypertension prevalence have been observed in sub-Saharan Africa, Oceania, and South Asia, in contrast to the more stable trends in high-income regions [9]. However, various health disparities have been reported, even in high-income countries. A Japanese national database study of 127.8 million participants revealed significant income-related inequalities in hypertension prevalence (40.2%/48.6% vs. 21.5%/33.3% in low-income vs. high-income women/men, respectively), suggesting socioeconomic gradients according to income, even within a high-income country [61]. Racial/ethnic differences and neighborhood deprivation contribute to undiagnosed hypertension in multiethnic countries, such as the United States [62]. In the United States, social determinants mediated 33.0% of the black–white difference in uncontrolled BP, with low-income, low educational attainment, and disadvantaged neighborhoods being key contributors [63]. Racism is a direct determinant of hypertension risk. Interpersonal and structural racism have been associated with elevated postpartum BP [64]. The American Heart Association recognizes structural racism as a fundamental driver of cardiovascular health disparities [65].

The COVID-19 pandemic highlighted the interaction between social determinants and access to health systems during crises. During COVID-19-related social restrictions, BP elevations were observed [66, 67]. In addition, we reported that patients treated for hypertension refrained from regular medical visits due to lower household income during the 2020 social restrictions compared to higher-income patients (19.6% vs. 8.8%) [68]. These factors may be related to the recent real-world evidence indicating the association between COVID-19 and cardiovascular diseases [69].

Environmental changes can influence BP and cardiovascular disease risk in a wide range of populations. A multinational study of 567 cities across 27 countries, including Japan, reported that both extreme heat and cold (outside the 1st–99th percentiles) temperatures were significantly associated with increased cardiovascular mortality [70]. Air pollution is a significant risk factor for developing hypertension. Chinese studies have demonstrated that long-term exposure to particulate matter ≤2.5 μm (PM2.5) was associated with increased BP, with remarkable effects in individuals with baseline elevated BP [71], and that this association might be mediated through arachidonate metabolite pathways [72]. Traffic noise and air pollution reportedly increase cardiovascular risk through shared mechanisms, including sympathovagal imbalance, endothelial dysfunction, and vascular inflammation [73]. Exposure to volatile organic compounds contributes to the risk of hypertension [74, 75]. Environmental exposure appears to affect BP in the pediatric population [76].

Disasters are critical factors for hypertension. After the Great East Japan Earthquake in 2011, BP increased, partly due to subsequent lifestyle changes resulting from evacuation [77–79]. The association between volcanic smog and elevated BP has been suggested [80]. Furthermore, occupational stress also manifests as BP patterns. A BP reduction from weekdays to the weekend is greater in individuals with high job strain and high job demands [81]. Acute circadian misalignment due to work-related social jetlag can increase arterial stiffness and morning BP surge [82].

## Epidemiology of hypertension control

Among women and men with hypertension in 2019 worldwide, 41% and 51% were estimated to be undiagnosed, 12% and 11% were diagnosed but untreated, and 24% and 20% were treated but uncontrolled, resulting in BP control rates of only 23% and 18%, respectively [9]. The situation appeared better in high-income Western and Asia-Pacific regions, where 27–34% of both women and men were undiagnosed, 10–14% were diagnosed but untreated, 21% were treated but uncontrolled, and the proportion of patients with controlled BP was 31–43%. The most concerning increases in the prevalence of hypertension were observed in sub-Saharan Africa, Oceania, and parts of South Asia, where BP control rates remain critically low.

Although Japan belongs to the high-income Asia-Pacific region and has a well-developed insurance system due to the Universal Health Insurance System, BP control in Japan seems to be suboptimal [9]. Among Japanese patients undergoing antihypertensive treatment, the proportion of patients with systolic/diastolic BP < 130/ < 80 mmHg was estimated to be 22.7–52.6% [83–87]. Our previous study reported that inadequate treatment and elevated pre-treatment BP levels were the two strongest factors for uncontrolled BP in Japan; 40.6% of cases with BP ≥ 140/ ≥ 90 mmHg and 25.6% of cases with BP ≥ 130/ ≥ 80 mmHg were due to failure to intensify antihypertensive medication to three or more agents [83]. These proportions could represent the quantifiable magnitude of “clinical inertia”, which refers to the failure of healthcare providers to initiate or intensify therapy despite unachieved therapeutic goals [88]. A Dutch cohort study identified BP levels closer to the target as factors associated with physicians being less likely to change antihypertensive therapy despite uncontrolled BP [89]. Furthermore, the recent report indicates that regional disparities in BP control exist even within Japan, revealing that healthcare resources significantly influence outcomes [90]. Even within a single country, comprehensive policy approaches encompassing the equitable distribution of healthcare resources may become crucial.

A hypertension-specialized clinic in Ohasama, where home BP was used to guide antihypertensive medication adjustment, achieved 93.6% home morning BP < 135/ < 85 mmHg between 2016 and 2019 [91]. Stroke incidence has shown a decreasing trend in Ohasama Town, Hanamaki City, Japan, where a community-based home BP monitoring program is ongoing as part of the Ohasama study [92]. These previous reports may provide an illustrative example of achieving target BP using home BP measurements [91, 92]. Also given that home BP measurements themselves can reduce BP [93–96], making home BP measurement devices more accessible would be beneficial. Further digitalization could be a key approach to achieving this, in part, through the development of wearable devices [97]. However, at present, healthcare providers should pay attention to the accuracy of BP measurement devices used by patients because unvalidated BP measurement devices are available for general consumers [98, 99]. A recent cross-sectional survey in Taiwan demonstrated that physicians identified device unreliability as a major barrier to the implementation of home BP measurement [100].

Medical teams, including non-physicians, are key factors in achieving good BP control [101]. A meta-analysis showed that pharmacist- and community health worker-led interventions produced the greatest BP reductions compared to physician-led interventions [102]. Another meta-analysis reported that multidisciplinary team-based care consistently outperformed usual care in controlling BP [103]. A large-scale Chinese cluster-randomized trial showed that when trained non-physician providers initiated and titrated antihypertensive medications following a stepped-care protocol under physician supervision, systolic/diastolic BP decreased by 23.1/9.9 mmHg, leading to a 33% reduction in the primary composite cardiovascular outcome [104].

## Future directions of hypertension epidemiology using real-world data

Research using real-world data, i.e., routinely collected data in regular practice [105, 106], is growing in the field of hypertension epidemiology. In Japan, insurer-based databases that integrate annual health checkups and claims data have been widely used as real-world data [66, 83, 107]. It has provided real-world associations between BP levels and cardiovascular or kidney diseases [83, 108–112] and information on actual healthcare patterns as partly introduced above [101, 110, 113, 114]. Classical epidemiological approaches, represented by well-designed prospective cohort studies, are recognized as possible data sources contributing to real-world evidence [105, 106] and have the potential to enhance its scientific rigor through the provision of novel information.

**Fig. 2:**
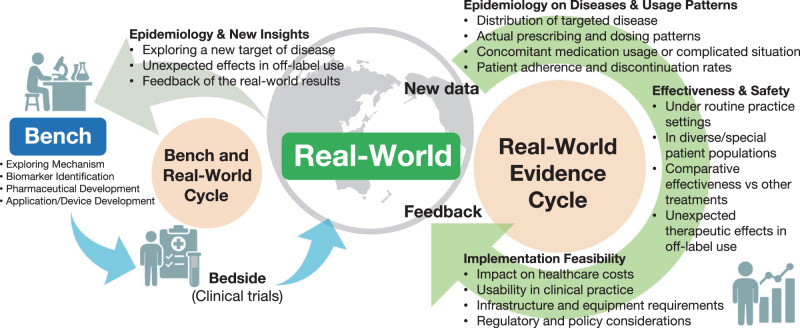
Real-world centered research cycles in modern hypertension epidemiology evolution from the traditional bench-to-bedside approach to real-world research paradigms. The framework includes bidirectional “Bench and Real-World Cycle” and continuous “Real-World Evidence Cycle” for optimizing hypertension interventions in clinical practice

Considering the broad availability of antihypertensive medications and BP measurements, real-world evidence will become increasingly important for advancing hypertension epidemiology and the social implementation of effective strategies to prevent and manage hypertension. Real-world evidence can be applied in practice, creating a feedback cycle back to the real world. Considering this, we propose the “Real-World Evidence Cycle” (Fig. 2), which will be a key concept describing the feedback cycle of usage patterns, effectiveness and safety information, and implementation feasibility from real-world data back to real-world practice. Additionally, the term “Bench to Bedside” has traditionally been used in translational research [115], but it could evolve into “Bench and Real-World Cycle” (Fig. 2).

The Real-World Evidence Cycle is a framework centered on the concept of real-world evidence. It emphasizes the importance of evidence derived from real-world data and the necessity of applying that evidence back to real-world settings. In this cycle, both the sources and applications of knowledge exist in the real world, creating a continuous process of evidence generation, validation, and implementation. By accelerating this circulation, the relationships between exposure and outcomes can be clarified in near real time, allowing rapid refinement of prevention and management strategies. The rapid report of BP elevation during the COVID-19 pandemic in the Japanese population exemplifies the value of timely real-world evidence [66], providing crucial early warnings that inform public health responses in Japan [116]. In this context, Japan’s advantage lies in its universal health insurance system, which enables the integration of nationwide health check-up data with medical claims databases. This comprehensive coverage allows for the creation of population-wide Real-World Evidence Cycle across diverse Japanese communities. However, reducing the barriers to real-world data access is crucial for maximizing this potential. For example, the National Health and Nutrition Examination Survey (NHANES) in the United States and Clinical Practice Research Datalink (CPRD) in the United Kingdom have relatively low barriers to data access, enabling their widespread use by researchers globally and accelerating evidence generation.

The “Bench and Real-World Cycle” complement this framework by integrating laboratory and interventional research within the same real-world system. Evidence obtained from real-world data can generate new hypotheses for mechanistic or experimental studies (“real world to bench”), while findings from these studies can be re-evaluated and contextualized under real-world conditions (“bench to real world”). This concept also incorporates the reverse process, referred to as “bedside to bench.” The term “Real-World” is broader and more comprehensive than “Bedside,” because modern healthcare is not confined to traditional clinical settings. It encompasses situations such as medication use outside hospitals and clinics, as well as healthcare devices operated at home or in community environments. Furthermore, the Real-World Cycle and the Bench and Real-World Cycle are particularly valuable in contexts where randomized clinical trials are ethically or practically infeasible, for example, studies on medication discontinuation or treatment in vulnerable populations such as pregnant women. Ultimately, promoting these real-world concepts encourages researchers to recognize that evidence generation must be coupled with its application in practice beyond bedsides, thereby bridging the gap between scientific discovery and population health improvement. Regarding the evaluation of treatment, target trial emulation (TTE), as proposed by Hernán et al., is a useful design principle that uses observational data to mimic randomized trials using real-world data [117, 118].

Considering the increasing importance of the “Real-World Evidence Cycle,” improving the accuracy and validity of real-world data is also an important challenge [107]. For example, despite the existence of established guidelines, only a small proportion of Japanese health checkup organizations routinely perform BP measurements in accordance with the recommendations [119, 120]. In China, the real-world office systolic BP differs from the unobserved automated office systolic BP by +18 mmHg [121]. The proportion of patients with controlled BP varies according to the measurement time and season [122–124]. Furthermore, the electronic health records used to define outcomes in research studies are often inaccurate because clinicians register the disease name or code on claims to receive reimbursement, leading to biased risk estimates [107]. At present, these limitations should be carefully considered and transparently reported when using real-world data from electronic health records. Classical epidemiological studies that systematically collect validated information on exposure and outcomes are indispensable for strengthening real-world evidence. The integration of digital hypertension technologies could further accelerate evidence generation [99], particularly when combined with efforts toward data standardization and institutional collaboration. Furthermore, the use of generative artificial intelligence may enable rapid analysis of standardized BP data at unprecedented scales, potentially transforming future hypertension management.

Table 2 shows perspectives on hypertension epidemiology in Asian populations. Among Asian populations, BP exerts a particularly strong influence compared with other metabolic risk factors [125]. Given the aging population, the importance of hypertension management in Asian populations is expected to increase.

**Table 2:** . Perspectives on hypertension epidemiology in Asian populations

Key points in Asians
• Lower-income countries generally exhibit a higher BP burden with limited control rates
• Even in high-income countries such as Japan, BP control remains suboptimal despite universal health coverage
• Substantial within-country regional variations in BP levels and control rates exist
• Blood pressure has a stronger impact relative to other metabolic risk factors for cardiovascular outcomes, particularly stroke
• Urinary sodium-to-potassium ratio is emerging as a practical biomarker for population-based risk assessment
• High salt sensitivity, potentially attributed to relative aldosterone excess, characterizes Asian populations
• PM2.5 air pollution significantly contributes to BP elevation
• Extreme temperatures and climate variability affect BP levels and cardiovascular mortality
• Gut microbiota dysbiosis is increasingly recognized as a contributor to hypertension pathogenesis
• Natural disasters (earthquakes, tsunamis) necessitate preparedness for post-disaster BP management
• Implementing home BP measurements may be a key strategy for improving BP control
• Real-world data integration offers opportunities for evidence-based interventions

## Conclusions

Hypertension is still a global public health challenge that requires immediate attention. Preventing hypertension effectively requires a comprehensive approach that addresses traditional individual-level risk factors, upstream social determinants, including socioeconomic disparities, environmental exposures, and structural racism, which profoundly influence hypertension. These considerations underscore the urgent need for innovative intervention strategies that extend beyond clinical settings and encompass community-based multidisciplinary approaches. Given this viewpoint, promoting a comprehensive “Bench and Real-World Cycle” and “Real-World Evidence Cycle” framework may become increasingly important in hypertension epidemiology. This will accelerate with the development of digital devices, including the evolution of home BP measurement devices and integration of electronic data. This will ultimately inform evidence-based policy decisions and optimize population-level interventions for this modifiable yet inadequately controlled cardiovascular risk factor.
